# Physical activity levels of an inpatient paediatric population: a cross-sectional service evaluation

**DOI:** 10.1136/bmjpo-2025-004179

**Published:** 2026-07-14

**Authors:** Colin Hamilton, Kieren Lock, Jonathan Littlewood, Sarah Nethercott, Theofilos Polychronakis

**Affiliations:** 1 University of Cambridge, Cambridge, UK; 2 Department of Physiotherapy, Cambridge University Hospitals NHS Foundation Trust, Cambridge, UK; 3 Norfolk and Norwich University Hospital NHS Trust, Norwich, UK; 4 Addenbrooke’s Hospital, Cambridge University Hospitals NHS Foundation Trust, Cambridge, UK

**Keywords:** Children, Resuscitation

## Introduction

 Physical activity (PA) is fundamental to healthy childhood development and is associated with benefits across physical, emotional, cognitive and social domains.^1^ A recent systematic review of PA levels in children in inpatient hospital settings identified a significant reduction in PA levels in children admitted with physical health conditions, though there was a limitation in the number of conditions that were reviewed.^2^ To address this, more real-world data is required. To understand the PA levels of the children admitted to a large tertiary hospital, a service evaluation was undertaken. This report describes the project.

## Methods

A cross-sectional observational service evaluation collected data on children’s PA levels. A random number generator was used to select a random subset of children between 7–16 years of age, admitted to one hospital over a 6-month period with no restrictions from their surgical or medical team, deemed to be well enough by an experienced children’s physiotherapist, had their PA levels measured with accelerometry.

Raw accelerometer data were processed using R. Daily step counts and time spent in varying intensities of PA were collected. Data were excluded from analysis for any day with less than 10 hours of recorded accelerometer wear time. Demographic, clinical characteristics and PA levels were analysed using proportions for categorical variables and measures of central tendency for continuous variables.

## Results

A total of 79 families were approached, of which 22 declined (34.18%). A pragmatic limit was placed on the number of families approached, limited by clinician availability. Demographics for the full data set are presented in table 1.

**Table 1:** Patient demographics

N	52
Female	21 (40.38%)
Mean (SD), age (years)	12.3 (2.6)
Median (IQR) length of stay prior to inclusion (days)	4 (4.5)
Median (IQR) length of stay post-inclusion (days)	1 (6.75)
Median (IQR) total length of stay (days)	8 (12.5)
Median (IQR) length of wear (days)	2 (2)
Median (IQR) length of wear per day (hours)	14.22 (4.17)
Median (IQR) length of wear total (hours)	30.05 (37.93)
Total group days of wear	145
Admitting specialty	
Oncology	16 (30.77%)
Respiratory	4 (7.69%)
Neurology	6 (11.54%)
Paediatrics	3 (5.77%)
Orthopaedics	2 (3.85%)
Surgical	10 (19.23%)
Gastroenterology	4 (7.69%)
Neurosurgical	7 (13.46%)

**Figure 1:**
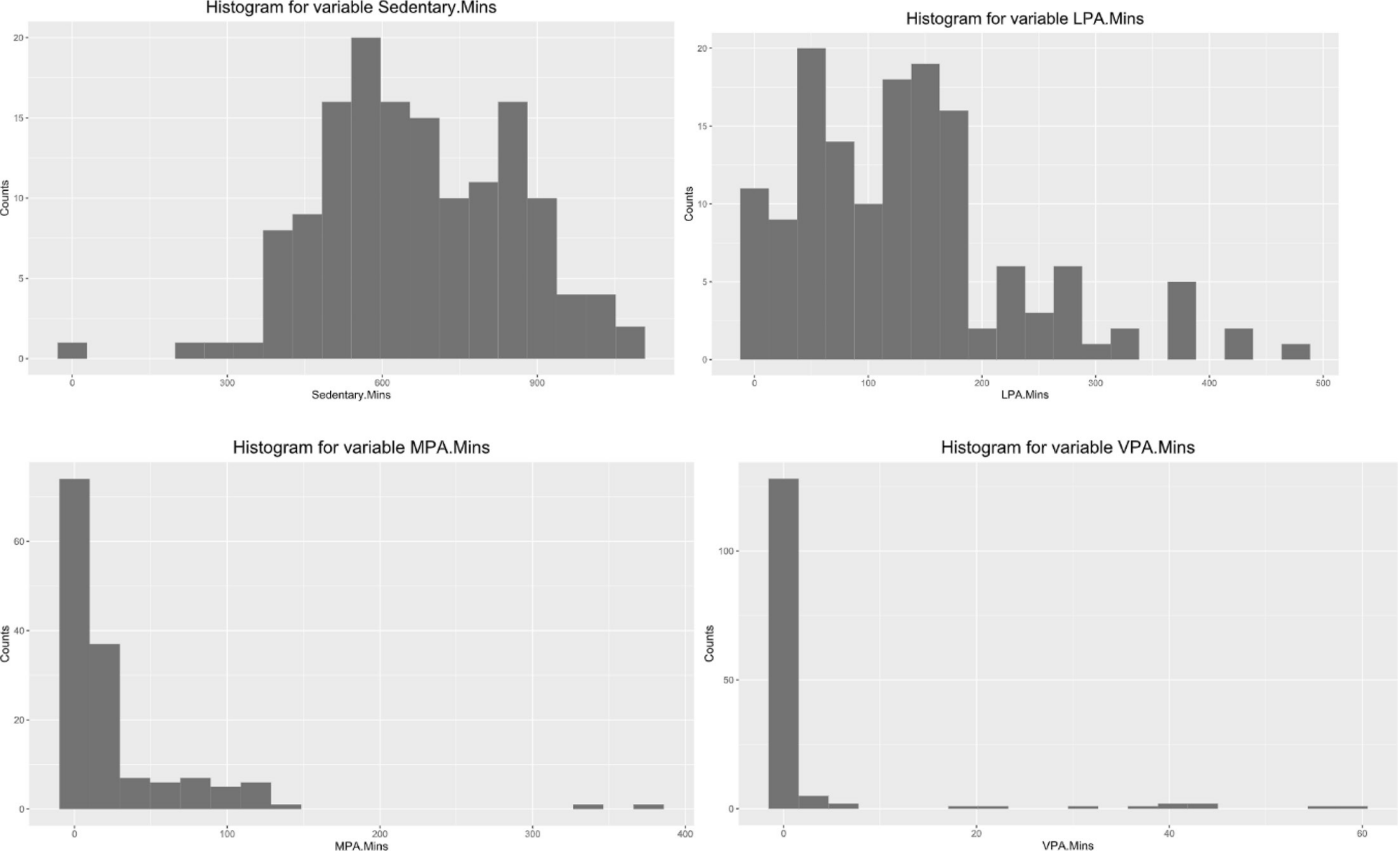
Most participants accumulate between 500 and 900 min of *sedentary time* per day. *LPA* shows more variability across individuals. Both *MPA* and *VPA* show only a small number achieving any time in these states. LPA, light physical activity; MPA, moderate physical activity; VPA, vigorous physical activity.

PA levels were consistently low, with a median (IQR) step count=766 (1992.00). Children spent the vast majority of wear time in sedentary behaviour with mean (SD) minutes in sedentary behaviour in a 24-hour period=668.48 (187.25). Children undertook a median (IQR) 123.00 (124.00) minutes of light PA (LPA), 9.00 (27.00) minutes in moderate PA (MPA) and 0.00 (0.00) in vigorous PA (VPA). However, a small number of children achieved higher levels of vigorous PA (figure 1).

A Spearman’s rank correlation investigated the relationship between time since admission and PA levels. While there was a significant relationship between day since admission and step count, the correlation was small and negative (r(142)= −0.22, p=0.00963), as was the association with time in MPA (r(142)=-0.201, p=0.01). There was no relationship between day since admission and time spent in any of sedentary (r(142)=0.16, p=0.052), LPA (r(142)=0.10, p=0.244) or VPA (r(142)=-0.137, p=0.102). This indicates that, in this group, children who had been in hospital longer were not more active than those who were recently admitted.

## Discussion

This study provides objective data on PA levels in hospitalised children. As the project is a service evaluation of one centre and a relatively high number of families declined, the conclusions that can be drawn and the generalisability of the study is limited. However, PA was strikingly low, in keeping with previous reports.^2^ Most participants in this study fell well below recommended thresholds of 60 min of moderate or vigorous PA a day or 20 min in children experiencing disability.^3^ However, it should be noted that these recommendations are not specific to the hospital environment. While there is almost certainly an influence of ill health on these data, it should be noted that all the children who participated had no restrictions placed on them by their medical or surgical team and had been assessed as able to be active by an experienced children’s physiotherapist. This raises the possibility of a primary impact of hospitalisation or the hospital environment itself on PA levels and supports concerns regarding deconditioning during hospital stays in adults and children.^4^ This also aligns with broader concerns that hospital culture often prioritises bed rest and passive recovery over active movement or rehabilitation, even when clinically appropriate.^5^
