# Supplementary material for: Physical activity levels of an inpatient paediatric population: a cross-sectional service evaluation
Source: BMJ Paediatr Open. 2026 Jul 14;10(1):e004179. doi: 10.1136/bmjpo-2025-004179 (PMC13374469; doi:10.1136/bmjpo-2025-004179)
Supplement: Supplementary file 1 [file bmjpo-2025-004179.reviewer_comments.pdf]

PEER REVIEW HISTORY

BMJ Paediatrics Open publishes all reviews undertaken for accepted manuscripts. Reviewers are asked to complete a checklist review form and are provided with free text boxes to elaborate on their assessment. These free text comments are reproduced below.

ARTICLE DETAILS

**TITLE (PROVISIONAL)**	Physical activity levels of an inpatient paediatric population: A cross-sectional service evaluation
**AUTHORS**	Hamilton, Colin<br>Lock, Kieren<br>Littlewood, Jonathan<br>Nethercott, Sarah<br>Polychronakis, Theofilos

VERSION 1 – REVIEW

**REVIEWER NAME**	Raj, Manu
**REVIEWER AFFILIATION**	Amrita Vishwa Vidyapeetham Amrita Health Sciences Campus, Pediatrics & Health Sciences Research
**REVIEWER CONFLICT OF INTEREST**	No Conflict of Interest
**AI DISCLOSURE**	No, I have not used AI
**DATE REVIEW RETURNED**	14-May-2026

**GENERAL COMMENTS**	1. The sample size of the study is shown as 52. Was this computed from any sample size estimation procedure ?<br>2. What random method was used to select participants?<br>3. The ethical clearance as well as informed consent is not mentioned in the manuscript. were these done and documented?<br>4. The study design is not mentioned anywhere in the manuscript.<br>5. The sample size is very low and any lack of association seen could be due to low power of the study to detect these associations. please bear this limitation in mind while discussing the findings.<br>6. The generalizability of the study findings are limited due to low response to study participation(52/79). This needs to be stated as well.

VERSION 1 – AUTHOR RESPONSE

Many thanks for your comments on our letter. I have attached the updated version. I have responded to your points as follows:

1. The sample size of the study is shown as 52. Was this computed from any sample size estimation procedure?

This was a pragmatic decision limited by clinician availability. This has now been stated in the manuscript.

2. What random method was used to select participants?

A random number generator was used. This has now been stated in the manuscript

3. The ethical clearance as well as informed consent is not mentioned in the manuscript. were these done and documented?

This was approved as a service evaluation by the hospital audit committee. I have clarified this in the manuscript.

4. The study design is not mentioned anywhere in the manuscript.

This was a prospective, cross-sectional, observational service evaluation. I have now stated this in the manuscript.

5. The sample size is very low and any lack of association seen could be due to low power of the study to detect these associations. please bear this limitation in mind while discussing the findings.

With this and the following point, I have included a statement at the beginning of the discussion highlighting the caution that should be taken in interpreting the data.

6. The generalizability of the study findings are limited due to low response to study participation(52/79). This needs to be stated as well.

Please see our response to point 5.
